# Successful Surgical Repair of Type A Acute Aortic Dissection in a Patient with Vascular Ehlers–Danlos Syndrome

**DOI:** 10.3400/avd.cr.21-00122

**Published:** 2022-03-25

**Authors:** Kousuke Mori, Hirohito Ishii, Shuhei Sakaguchi, Daichi Sakurahara, Ayaka Iwasaki, Koji Furukawa

**Affiliations:** 1 Department of Cardiovascular Surgery, Faculty of Medicine, University of Miyazaki, Miyazaki, Miyazaki, Japan

**Keywords:** vascular Ehlers–Danlos syndrome, type A acute aortic dissection, total arch replacement

## Abstract

Surgery for vascular complications of a patient with vascular Ehlers–Danlos syndrome (vEDS) is challenging due to the fragility of the associated tissues. In this study, we present a type A acute aortic dissection case in a patient with vEDS successfully treated via total arch replacement. A 42-year-old woman was transferred to our hospital 10 days after the onset of symptoms and underwent emergency surgery. Intraoperative findings revealed severe inflammatory changes without tissue fragility that is distinctive of vEDS. The postoperative course was uneventful except for left recurrent laryngeal nerve palsy, and 24 months after the operation, the patient has remained free from any arterial event.

## Introduction

Ehlers–Danlos syndrome (EDS) has been identified as an uncommon inherited disorder of connective tissues characterized by skin hyperextensibility, joint hypermobility, and tissue fragility.^1)^ Of the 13 subtypes of this syndrome, vascular EDS (vEDS) is deemed the most fatal because of the risk of arterial rupture and hemorrhage and intestinal or uterine rupture. Due to vessel fragility, a generally accepted strategy for dealing with vascular complications of vEDS is a conservative approach, with operative treatment reserved for patients presenting with imminent life-threatening bleeding.^2)^ Recent mechanistic investigations have revealed that the prognosis of patients with vEDS differs depending on the type of gene mutation,^3,4)^ and several successful cases of surgical treatment in patients with vEDS have been reported.^5–7)^ Herein, we present a case of type A acute aortic dissection in a patient with vEDS successfully treated by total arch replacement.

## Case Report

**Figure 1:**
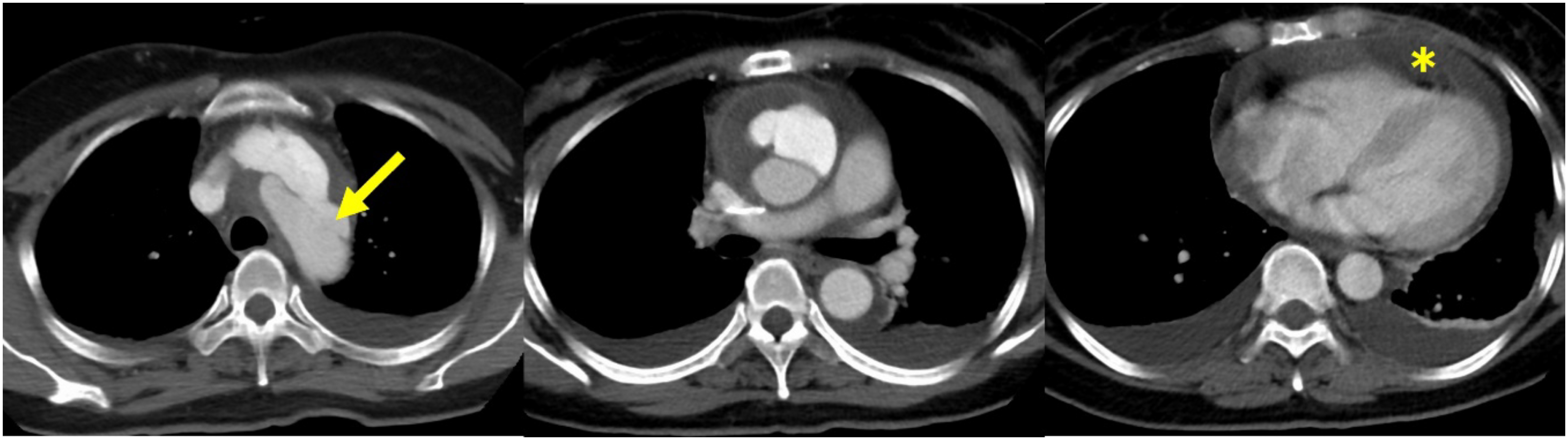
Fig. 1 Contrast-enhanced computed tomography image showing type A acute aortic dissection with patent false lumen with a maximum diameter of 32 mm, intimal tear in the aortic arch (arrow), and moderate pericardial effusion (asterisk). The diameter of dissecting aortic aneurysm of the ascending aorta was 53 mm in a maximum minor axis.

A 42-year-old woman with a history of hypertension was admitted to the hospital due to the sudden onset of left neck pain. Magnetic resonance imaging revealed bilateral internal carotid artery dissection. Two days after admission, she developed left hemiparesis and mild articulatory disorder due to acute cerebral infarction with right internal carotid artery obstruction. Ten days after admission, contrast-enhanced computed tomography (CT) was performed to examine palpitation revealed type A acute aortic dissection, and she was then transferred to our hospital. She had four pregnancies and childbirths without complications. Her family history included a younger brother and mother that had died of acute aortic dissection and a grandmother and great-grandmother that died suddenly. Her younger brother had a marked fragility of the aortic wall during the operation for type A acute aortic dissection at another hospital. The surgeon at the time described the aortic wall as if it was “tofu.” On physical examination, she had prominent eyes, thin lips, and a small chin, without hypermobility of small joints and hyperelastic skin, and with left hemiparesis. The contrast-enhanced CT revealed a type A aortic dissection with a patent false lumen and pericardial effusion (**Fig. 1**), and a head CT revealed a low-density area in the right frontotemporal lobe.

Although vEDS was suspected from her family history and physical findings, her present condition was considered significantly life-threatening due to aneurysm rupture or cardiac tamponade; thus, emergent total aortic arch replacement was performed. After a median sternotomy was performed, cardiopulmonary bypass (CPB) was established via the right femoral artery and bicaval venous drainage. Under hypothermic circulatory arrest at a bladder temperature of 25°C, the aortic arch was opened, and selective cerebral perfusion was then initiated. Detachment around the aortic arch and three vessels was difficult due to excessive adhesions. The arterial wall was not as fragile as seen in vEDS; however, it appeared to have thickened, likely due to inflammation. The aortic arch was reconstructed using a 22-mm woven Dacron graft (J Graft SHIELD; Japan Lifeline, Tokyo, Japan). The brachiocephalic artery could not be anastomosed due to severe inflammation, so the first branch of the graft was anastomosed to the right subclavian artery with an end-to-side fashion, and the proximal end of the brachiocephalic artery was closed. All anastomoses were created delicately and reinforced with Teflon felt, and aortic anastomoses were sealed with biological glue. Thorough replacement of blood factors and platelets was also required for hemostasis.

**Figure 2:**
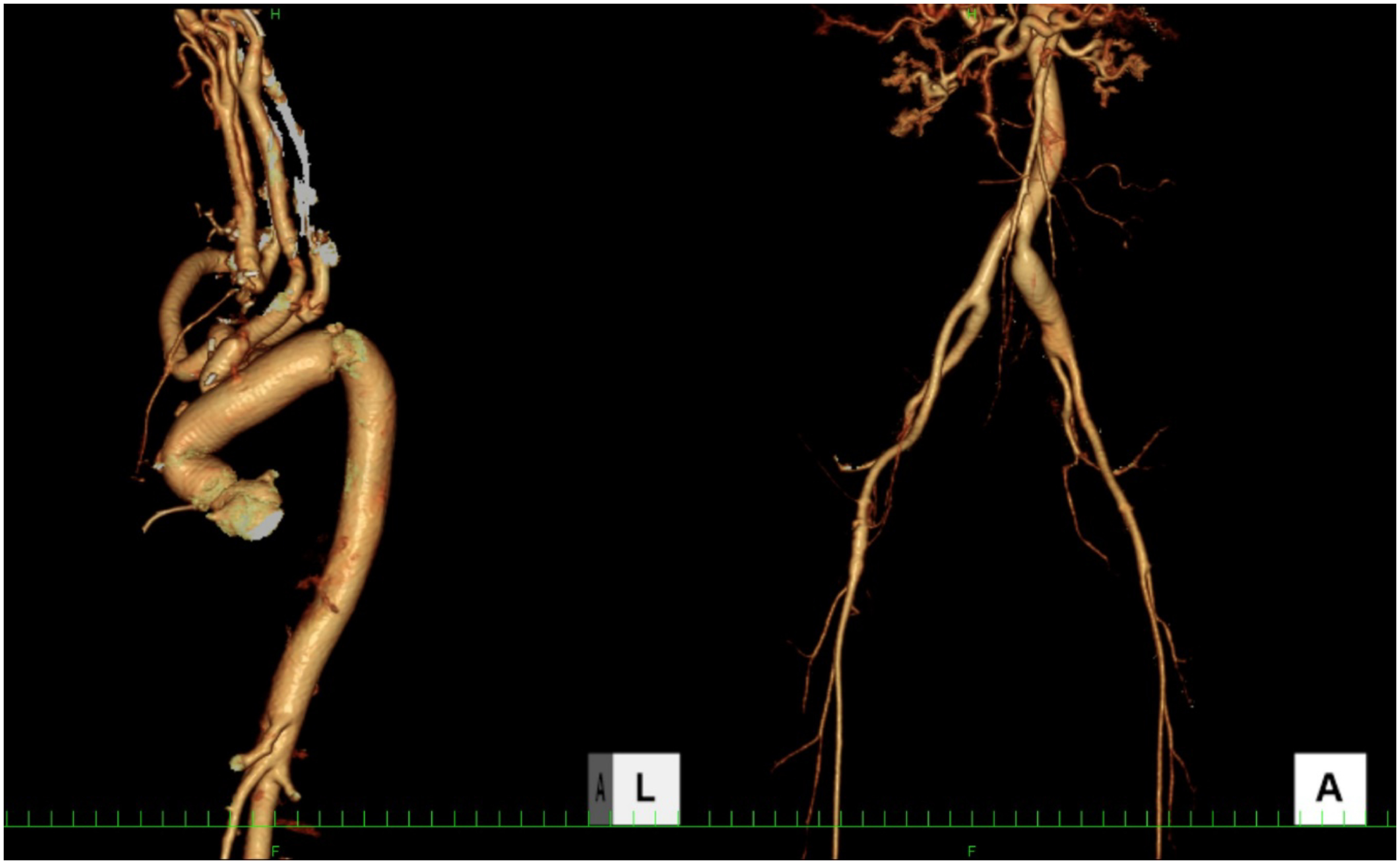
Fig. 2 Three-dimensional reconstruction of a postoperative computed tomography scan showing complete thrombosis of the false lumen without abnormal findings of anastomotic site and new aortic aneurysm or dissection.

The surgical duration was 611 min, the CPB duration was 335 min, and the total time for cardiac and circulatory arrest was 186 min and 72 min, respectively. The patient was then extubated on postoperative days 3 and 6; however, both days required reintubation because of respiratory failure and difficult sputum discharge. Although she could finally extubate on postoperative day 16, she was diagnosed with left recurrent laryngeal nerve paralysis. A genetic test revealed the mutation in the COL3A1 gene with a substitution for glycine in the triple-helical domain. She was diagnosed with vEDS because of the developed aortic dissection, family history, characteristic facial appearance, and gene mutation. Postoperative contrast-enhanced CT revealed complete thrombosis of the false lumen without abnormal findings of the anastomotic site and new aortic aneurysm or dissection (**Fig. 2**). Although she suffered from mild left upper limb paresis and hoarseness, she was transferred to a rehabilitation hospital without any new arterial complications. The patient remains well with no evidence of arterial events 24 months after the operation.

## Discussion

EDS has been identified as a rare autosomal dominant connective tissue disorder resulting from mutations in the COL3A1 gene encoding for type III collagen synthesis.^1)^ In 2017, a new classification of EDS was established. In this new classification, 13 subtypes were recognized, each with different complications and outcomes.^1)^ Vascular EDS is particularly rare with a prevalence of <1 in 100,000 and 4% of all EDS subtypes; however, it has been considered the most fatal form because of possible arterial rupture and hemorrhage and/or intestinal or uterine rupture. More than 80% of these patients experience a significant complication by the age of 40 years, and the median survival age was 48 years.^8)^ Besides, pregnancy for women with vEDS has an estimated mortality risk of 5.3%–6.7% due to peripartum arterial or uterine rupture.^8,9)^

The traditional recommendation for vascular complications associated with vEDS has been a conservative approach owing to the high operative mortality caused by vessel fragility.^2)^ Within 30 years of observing 31 patients from Mayo Clinic with vEDS, 70% of vascular operations were performed urgently or as emergency procedures, and the mortality rate of these operations was 47%.^2)^ Pepin et al. also reported high operative mortalities of 41% in cases of arterial complications.^3)^ Conversely, several case reports of successful surgical treatment of vascular complications in patients with vEDS have been published.^5–7)^

Pepin et al. demonstrated that the natural history of patients with vEDS varies with gender and type of gene mutation in COL3A1.^3)^ Additionally, Frank et al. demonstrated significant differences in terms of age at which the first significant event occurred between index patients with vEDS and their first-degree relatives.^4)^ These articles demonstrate that operative mortality and life expectancy in patients with vEDS vary widely.

A substitution for glycine in the triple-helical domain is the most common mutation in patients with vEDS. Pepin et al. analyzed the survival difference by gender and type of gene mutation and revealed that female and glycine substitution had a relatively good prognosis. Furthermore, they have also revealed a significant difference in survival depending on the type of amino acid resulting in the glycine substitutions.^3)^ Frank et al. analyzed the association between the age of onset of the first significant complication and type of gene mutation and revealed that glycine substitution had a later age at the onset of the first complication than a splice-site variant.^4)^ Based on the results of genetic test and her clinical characteristics, including late onset age of first complication, four pregnancies and history of childbirth without complications, and no fragility of the aortic wall, it is likely that our patient had the type of gene mutation that offers a relatively good prognosis.

Conversely, it is true that some patients have a poor prognosis. Therefore, when performing surgery on a patient with vEDS, thoughtful preparations, such as technical devising and securing sufficient blood products, are required to deal with bleeding. Thus, in this case, although the lack of tissue fragility may have led to the success of the surgery, we ensured other preparations as well.

However, diagnosis with vEDS is often difficult to be made before the presentation of complications. Indeed, only 26% of patients with vEDS can be diagnosed before presentation,^2)^ thus making it difficult to preoperatively recognize the type of gene mutation and infer the patient’s prognosis in clinical practice. Therefore, vascular surgeons must be aware that there are wide variations in tissue fragility that are associated with the type of gene mutations in patients with vEDS.

## Conclusion

The operative risk and prognosis of patients with vEDS vary widely from case to case. Thus, when a patient is in a life-threatening situation due to vascular complications, healthcare providers should perform surgical intervention considering gender, age of onset, and patient history. Furthermore, if it is possible to identify the type of gene mutation preoperatively, performing elective surgery for vascular complication indicated for intervention can be considered depending on the operative risk.

## References

[1] Malfait F, Francomano C, Byers P, et al. The 2017 international classification of the Ehlers–Danlos syndromes. Am J Med Genet C Semin Med Genet 2017; 175: 8-26. 28306229 10.1002/ajmg.c.31552

[2] Oderich GS, Panneton JM, Bower TC, et al. The spectrum, management and clinical outcome of Ehlers–Danlos syndrome type IV: a 30-year experience. J Vasc Surg 2005; 42: 98-106. 16012458 10.1016/j.jvs.2005.03.053

[3] Pepin MG, Schwarze U, Rice KM, et al. Survival is affected by mutation type and molecular mechanism in vascular Ehlers–Danlos syndrome (EDS type IV). Genet Med 2014; 16: 881-8. 24922459 10.1038/gim.2014.72

[4] Frank M, Albuisson J, Ranque B, et al. The type of variants at the COL3A1 gene associates with the phenotype and severity of vascular Ehlers–Danlos syndrome. Eur J Hum Genet 2015; 23: 1657-64. 25758994 10.1038/ejhg.2015.32 PMC4795191

[5] Conte S, Serraf A, Lacour-Gayet F, et al. Successful repair of thoracic aortic aneurysm in a child with Ehlers–Danlos syndrome. J Thorac Cardiovasc Surg 1997; 113: 410-1. 9040636 10.1016/S0022-5223(97)70339-8

[6] Babatasi G, Massetti M, Bhoyroo S, et al. Pregnancy with aortic dissection in Ehler–Danlos syndrome. Staged replacement of the total aorta (10-year follow-up). Eur J Cardiothorac Surg 1997; 12: 671-4. 9370418 10.1016/s1010-7940(97)00211-x

[7] Hamano K, Minami Y, Fujimura Y, et al. Emergency operation for thoracic aortic aneurysm caused by the Ehlers–Danlos syndrome. Ann Thorac Surg 1994; 58: 1180-2. 7944780 10.1016/0003-4975(94)90488-x

[8] Pepin M, Schwarze U, Superti-Furga A, et al. Clinical and genetic features of Ehlers–Danlos syndrome type IV, the vascular type. N Engl J Med 2000; 342: 673-80. 10706896 10.1056/NEJM200003093421001

[9] Murray ML, Pepin M, Peterson S, et al. Pregnacy-related deaths and complications in women with vascular Ehlers–Danlos syndrome. Genet Med 2014; 16: 874-80. 24922461 10.1038/gim.2014.53

